# Prevalence of gambling disorder and its correlates among homeless men in Osaka city, Japan

**DOI:** 10.1007/s10899-022-10121-x

**Published:** 2022-06-15

**Authors:** Chiyoung Hwang, Taichi Takano, Ryuhei So, Ethan Sahker, Sho Kawakami, Charles Livingstone, Naoko Takiguchi, Masako Ono-Kihara, Masahiro Kihara, Toshi A. Furukawa

**Affiliations:** 1 Department of Health Promotion and Human Behavior, Graduate School of Medicine, School of Public Health, Kyoto University, Yoshida-Konoe-cho, Sakyo-ku, 606-8501 Kyoto, Japan; 2 Not-for-Profit Organization, The Big Issue Japan Foundation, Osaka, Japan; 3 Department of Psychiatry, Okayama Psychiatric Medical Center, Okayama, Japan; 4 Gambling and Social Determinants Unit, School of Public Health and Preventive Medicine, Monash University, Melbourne, Victoria Australia; 5 Department of Sociology, Otani University, Kyoto, Japan; 6 Interdisciplinary Unit for Global Health, Center for the Promotion of Interdisciplinary Education and Research, Kyoto University, Kyoto, Japan; 7 Population Health and Policy Research Unit, Medical Education Center, Graduate School of Medicine, Kyoto University, Kyoto, Japan

**Keywords:** Gambling, Homelessness, Addiction, Prevalence, Associated factors

## Abstract

Internationally, the prevalence of gambling disorder has been reported to be higher among homeless people than the general population; however, little is known about the factors associated with gambling disorder in this population. The present study aimed to investigate the prevalence of gambling disorder and its associated factors among homeless men using shelters in Osaka City. A cross-sectional survey was conducted from 30 to 2018 to 4 January 2019, using the 2017 Japanese National Survey of Gambling (JNSG) questionnaire, supplemented with questions about homeless experiences, drinking, and smoking. Using the South Oaks Gambling Screen, the presence of gambling disorder was determined by a score ≥ 5 out of 20. Multivariate logistic regression was conducted to explore factors associated with lifetime gambling disorder. Lifetime and past-year prevalence of gambling disorder among 103 participants was 43.7% (95% confidence interval [CI]: 34.5–53.3) and 3.9% (95% CI: 1.5–9.6), respectively, which are higher than the 6.7% and 1.5% found among men in the 2017 JNSG. Reasons reported for currently gambling less were primarily financial. Factors associated with lifetime GD included “more than 20 years since the first incidence of homelessness” (adjusted odds ratio [AOR]: 4.97, 95% CI: 1.50–16.45) and “more than five incidences of homelessness” (AOR: 4.51, 95% CI: 1.06–19.26). When homeless individuals with gambling disorder try to rebuild and stabilize their lives, the presence or resurgence of gambling disorder may hinder the process and pose a risk of recurring homelessness. Comprehensive support services for homeless individuals with gambling disorder are required. (250 words)

**Supplementary information:**

The online version contains supplementary material available at 10.1007/s10899-022-10121-x.

## Introduction

With the global proliferation of commercialized gambling, it appears that homeless people are particularly vulnerable to gambling addiction. Despite differences between countries in which studies have been conducted, survey instruments, and targeted subgroups within the homeless population, the prevalence of gambling disorder (GD) or pathological gambling among homeless people ranges from 12 to 24.6% for lifetime incidence (Matheson et al., 2014; Nower et al., 2015), and 5.5–23.6% for past-year incidence (Shaffer et al., 2002; Sharman et al., 2015, 2016; Wieczorek et al., 2019). These rates far exceed those of the general population. For example, homeless people in the United Kingdom were 16 times more likely to have a gambling problem than the general population (Sharman et al., 2015).

However, risk factors for GD among homeless people and how these differ from those of the general population are not fully understood. In both homeless and general populations, being male and young are factors associated with GD (Johansson et al., 2009; Nower et al., 2015; Shaffer et al., 2002; Wieczorek et al., 2019). Yet, factors such as comorbid psychiatric disorders—including alcohol and substance use disorders—the form and frequency of gambling, the number and duration of homelessness experiences, and negative life events experienced prior to homelessness have only been identified in a single study or shown conflicting results in different studies (Nower et al., 2015; Shaffer et al., 2002; Sharman et al., 2016; Wieczorek et al., 2019). For instance, Nower et al. (2015) reported that in a homeless sample of predominantly African American men in the United States, those with problem gambling were more likely to meet the diagnostic criteria of bipolar disorder, post-traumatic stress disorder (PTSD), antisocial personality disorder (ASPD), and nicotine, alcohol, or other substance abuse or dependence than those without. However, Sharman et al. (2016) found no association between the high prevalence rate of alcohol dependence (23.6%) and other substance dependence (31.9%) among their participants and gambling problems. Furthermore, among homeless people in the United State participating in substance use disorder programs, those with more severe gambling problems tended to be homeless more often and at younger ages (Shaffer et al., 2002), while other studies have found no such trend (Nower et al., 2015; Wieczorek et al., 2019). Regarding the pre- and post-temporal relationship between gambling problems and homelessness, only Sharman et al. (2016) reported that in 82.4% of problem gamblers, gambling problems preceded homelessness.

Gambling is technically illegal in Japan. However, some forms of gambling, such as lotteries and horse, bicycle, boat, and motorbike racing, are allowed as “public gambling.” Moreover, “Pachinko” and “Pachislot” (electronic gaming machine [EGM]) parlors are ubiquitous across Japan and easily accessible. Internationally, EGMs are recognized as an important gambling form; however, legal loopholes in Japan render EGMs acceptable. Gamers win tokens, which are non-monetary prizes, and tokens are then sold to third-party vendors for cash equivalent values. According to the 2017 Japan National Survey of Gambling (JNSG, 2017), the prevalence of GD in the general population was 3.6% and 0.8% for lifetime and past-year, respectively (6.7% and 1.5% among men, respectively) with approximately 80% of people with GD spent their money mainly on EGMs (Higuchi & Matsushita, 2017).

There are currently no methodologically valid epidemiological studies, and only fragmented reports have focused on GD among homeless people in Japan. The findings of a comprehensive nationwide survey indicated that 1.1–10.3% of homeless people were recognized by service providers as engaging in problem gambling (Specified Non-profit Organization National Homeless Support Network, 2011). Moreover, in the national survey on the conditions of the homeless, 8.8% of respondents reported “drinking and gambling” as their reason for living on the streets (Ministry of Health, Labour and Welfare, 2016). Further, a study of cognitive dysfunction with a sample of 16 homeless men in Tokyo found that five met the criteria of “pathological gambling” (Pluck et al., 2015). Considering these reports, homeless people are likely a high-risk population for GD in Japan. However, Japan does not recognize GD among homeless people as a public health concern; therefore, there is no support system to adequately address these concerns. To develop prevention, intervention, and support measures for homeless people with GD, it is necessary to investigate the prevalence of GD and its characteristics among this population.

The present study aims to provide initial epidemiological information on homelessness and GD in Osaka, Japan’s second largest city, where one of the best-known shelters for homeless people is located. First, we will describe prevalence of potential GD and the situation of gambling activities among homeless men staying in shelters in Osaka City. Second, we will investigate associated factors between homelessness and GD.

### Methods

### Study Design and setting

We conducted a cross-sectional survey between December 30, 2018 and January 4, 2019, in Osaka City. Osaka City has the largest homeless population in the country as well as Tokyo. According to the Japanese National survey on “the actual conditions of the homeless 2019,” which is conducted every year by the Ministry of Health, Labour and Welfare, 4,555 homeless people were visually identified in Japan’s streets, parks, train stations, riversides, and other facilities in 2018, and of them, 1,002 (981 men and 21 women) were identified in Osaka City, including those who made use of free public shelters (Ministry of Health, Labour and Welfare, 2019).

The free public shelters, which constitute the study setting, are located in an area with the highest number of homeless people in Osaka City. This area is the largest gathering place of day labor recruiters and job seekers in western Japan, called “Yoseba,“ and many cheap simple accommodations for day laborers are available here. Due to the recession and aging of the day laborers, many day laborers become homeless. This area has many public and private support services not only for such homeless people but also for other economically and socially vulnerable individuals.

The free public shelters provided by the Osaka City government are available for only men and ordinarily allow for a one-night stay. During this survey, the Osaka City government conducted a social welfare program for homeless people, the Emergency Accommodation Support Project (EASP), which offered accommodation and meals for six consecutive days around the New Year. Those who applied for EASP had to consult and register with the local municipality. Those needing care due to poor health or advanced age were preferentially placed in a care center, while others were placed in standard shelters. In 2018, 344 individuals registered with the EASP. Among these, all 265 homeless men staying in shelters (age rang = 26–83 years [M ± SD = 60.5 ± 8.1]; monthly income range = ¥0–160,000 [M ± SD = 38,684 ± 30,275, median: 30,000]) were potential participants in this study.

### Procedure

During the EASP, all shelter users were informed about the study, including that they could not participate if they were intoxicated. Posters, which contained all the information, were put up on the walls of the shelters. Research staff orally requested research cooperation at every lunch meal and distributed flyers each bed. Those who were willing to participate in the survey registered in front of the shelters and were given an invitation stating their full-name, and details of the survey: purpose, contents, date, and time and location of the survey. Data were collected individually at a nearby interview office, a 5-minute walk from the shelters, to protect participants’ privacy. Each survey took approximately 40–60 min. Participants were remunerated with ¥1,000 for participating.

### Measures

We used the structured questionnaire of the 2017 Japan National Survey of Gambling (JNSG 2017), which was conducted by the Kurihama Medical and Addiction Center, commissioned by the Japan Agency for Medical Research and Development. The questionnaire included basic demographic information such as age, educational qualifications, marital status, and estimated annual income during the current and most frequent gambling periods, as well as questions about gambling-related experiences, screening of problem gambling, risk perception, and help-seeking behaviors related to gambling problems. In the questionnaire of the JNSG 2017, gambling frequency and annual income were assessed as multiple-choice responses. This was supplemented with questions about homelessness experiences (e.g., duration between the first incidence of homelessness and the date of the survey, number of homelessness experiences, use of public social resources), smoking status, and alcohol use.

The South Oaks Gambling Screen (SOGS; Lesieur & Blume, 1987) was used to determine potential GD. It consists of 20 items; items with two choices are scored 0 for *not applicable* and 1 for *applicable*; items with three choices are scored 0 for *not applicable*, and 1 for all others; and items with four choices are scored 1 only if the two most frequent choices are selected. Possible total scores range from 0 to 20, and following the JNSG criteria, a score of ≥ 5 indicates potential GD. In the present study, the Cronbach’s alpha coefficient of the Japanese version of SOGS (Kido & Shimazaki, 2007; Saito, 1996) was 0.88 for lifetime prevalence and 0.68 for the past year.

The Problem Gambling Severity Index (PGSI; Ferris & Wynne, 2001) was used to identify problem gambling risk in the past year. The PGSI consists of nine items scored as *never* = 0, *sometimes* = 1, *most of the time* = 2, and *almost always* = 3. Total scores range from 0 to 27 and are categorized according to the JNSG criteria as 0 = *no risk*, 1–2 = *low risk*, 3–7 = *medium risk*, and 8–27 = *high risk*. The Cronbach’s alpha coefficient of the Japanese version of the PGSI (So et al., 2019) in the present study was 0.80.

The Alcohol Use Disorders Identification Test (AUDIT) was used to assess the risk for alcohol use disorder (Babor et al., 2001). The AUDIT consists of 10 items regarding alcohol use, alcohol use disorder symptoms, and alcohol-related problems. Each item is scored on a scale of 0–4, with overall scores ranging from 0 to 40. We followed the Japanese criteria: a score of 0–7 = *no problem*, 8–15 = *hazardous use*, and 16–40 = *potential alcohol dependence*. The Cronbach’s alpha coefficient of the Japanese version of the AUDIT (Hiro & Shima, 1996) for the present study was 0.90.

The order in which the questionnaires were presented is as follows: (1) demographic information, (2) homelessness history, (3) gambling experiences, (4) PGSI, (5) SOGS, (6) help seeking behaviors, (7) smoking status, and (8) alcohol use.

### Statistical analysis

First, descriptive analyses were performed using the participants’ sociodemographic characteristics and their gambling experiences. We then recorded the characteristics of their gambling activities for the period when they had gambled the most and during the past year. We categorized the data and described the distribution by age, time elapsed since the first incident of homelessness, number of homelessness experiences, gambling frequency, age at onset of gambling, monthly and annual income (as of December 2018), PGSI scores, and AUDIT scores. As public gambling is illegal for those under the age of 20 in Japan—except for Pachinko and Pachislot, which are allowed from the age of 18—we categorized the age of gambling initiation as “< 20 years” and “≥ 20 years.” We examined whether the continuous variables were normally distributed through histograms and the Shapiro-Wilk test. We conducted the Mann-Whitney U test as a sensitivity analysis where possible.

Second, we used univariable and multivariable logistic regression analyses to explore factors associated with potential GD according to the criteria of SOGS. As this is the first investigation on GD among homeless people in Japan, a two-step exploratory approach was conducted. In the first step, bivariate analyses and chi-square tests were performed to investigate the cross-sectional associations between “potential GD (lifetime)” and each independent variable. The odds ratio (OR), 95% confidence interval (CI), and *p*-values were calculated. Second, to identify strong correlates of lifetime GD, we simultaneously entered the following characteristics in a multivariable logistic regression: a priori selection of well-established risk factors for GD (Johansson et al., 2009), including young age (< 30 years old), smoking, alcohol dependence, and imprisonment history as well as the variables that were associated with *p* ≤ .10 in the first step; we then selected only those that maintained a significance of *p* ≤ .10. The adjusted OR, corresponding 95% Cis, and *p*-values were calculated. The *p*-values were two-sided with a significance level of 0.05. Finally, we conducted a sensitivity analysis, in which we entered all the covariates. Statistical analyses were performed using JMP Pro14 (SAS Institute Inc. Cary, NC, USA).

### Ethics

This study was conducted in accordance with the Declaration of Helsinki. Ethical approval was obtained from the institutional review board of the second author’s institution and the ethics committee of the first author’s institution. We informed the participants about the study procedures and that participation was entirely voluntary. There were no disadvantages associated with refusing participation, and participation could be withdrawn at any point during the survey. We obtained written informed consent.

## Results

### Participants

Of the 265 homeless men staying in shelters, 112 (42.3%) voluntarily participated in this survey. The final number of valid respondents was 103 (38.9%). Nine participants were excluded due to inconsistent answers regarding the time since the first incidence of homelessness, the number of homelessness experiences, and their social support usage. Table 1 and Supplementary File 1 show participant demographics and other characteristics. The mean age of the participants was 58 years (SD = 8.5, median = 58, range = 30–73), and 89.3% (*n* = 92) participants earned an income through some type of work, such as relief unemployment work provided by Osaka City municipality, daily labor work, and/or aluminum can collection. The median monthly income was ¥40,000 (M ± SD = 49,229 ± 37,992, range = 0–150,000).

**Table 1:** Demographic and other characteristics of participants (*N* = 103)

	*n*	(%)
**Age (years)**		
≤ 39	2	(1.9)
40–49	16	(15.5)
50–59	41	(39.8)
60–69	39	(37.9)
≥ 70	5	(4.9)
**Marital status**		
Never married	74	(71.8)
Divorced	24	(23.3)
Unmarried partner	3	(2.9)
Widowed	2	(1.9)
**Educational qualifications** ^**a**^		
Middle school graduate	54	(52.4)
High school graduate	37	(35.9)
Graduate from a higher education institute	12	(11.7)
**Time elapsed since the first homelessness incidence**		
< 1 year	9	(8.7)
1 year–less than 5 years	11	(10.7)
5 years–less than 10 years	23	(22.3)
10 years–less than 20 years	30	(29.1)
20 years–less than 30 years	20	(19.4)
≥ 30 years	10	(9.7)
**Number of homelessness episodes in lifetime**		
1	49	(47.6)
2–4	37	(35.9)
≥ 5	17	(16.5)
**Experience of using social support in lifetime (multiple answers allowed)**		
Welfare: home	47	(45.6)
Welfare: facility	16	(15.5)
Independent living support facilities	28	(27.2)
**Income in December 2018 (¥)**		
None	11	(10.7)
< 30,000	15	(14.6)
30,000–59,999	42	(40.8)
60,000–89,999	15	(14.6)
90,000–119,999	12	(11.7)
≥ 120,000	8	(7.8)
**Current smoking behaviour**		
Yes	77	(74.8)
No	26	(25.2)
**Current alcohol use (AUDIT score)**		
Potential alcohol dependence (15–40)	17	(16.5)
Hazardous use (8–14)	15	(14.6)
No risk (1–7)	36	(35.0)
Non-drinker (0)	35	(34.0)
**Imprisonment history in lifetime**		
Yes	17	(16.5)
No	86	(83.5)

¥: Japanese Yen

AUDIT: Alcohol Use Disorders Identification Test

^a^In case of dropout, educational qualifications were classified as the preceding educational level (14 high school dropouts and three college dropouts)

### Gambling experience and the prevalence of potential gambling disorder

Data on gambling experience and GD are shown in Tables 2 and 3, and Supplementary File 1. According to the SOGS criteria, prevalence of lifetime potential GD was 43.7% (*n* = 45), and past-year prevalence was 3.9% (*n* = 4). Further, 7.8% (*n* = 8) met the criteria for high-risk gambling (PGSI: 8–27 points) in the past year. Among the 45 participants with potential GD (lifetime), 17.8% (*n* = 8) were high-risk gamblers, 35.6% (*n* = 16) were moderate-risk gamblers, and 15.6% (*n* = 7) were low-risk gamblers according to PGSI criteria. For those who gambled, the median age at which they started was 18 years (M ± SD = 19.5 ± 6.37, range: 10–56) and their first form of gambling was Pachinko and Pachislot (71.1%). Of the participants, 88.7% (*n* = 86) stated that they no longer gambled as much as they did previously, primarily because they did not have enough money (*n* = 46).

**Table 2:** Gambling experience, prevalence of gambling disorder, and severity of gambling risk among the participants

	*n*		(%)
**Gambling experience (*N*** **= 103)**			
In lifetime	97		(94.2)
In the past year	71		(68.9)
**Potential GD (SOGS score ≥ 5) (*N*** **= 103)**			
In lifetime	45		(43.7)
In the past year	4		(3.9)
**Severity of gambling risk in the past 12 months (PGSI score) (*N*** **= 71**^**a**^**)**			
High-risk gambler (8–27)	8		(11.3)
Moderate-risk gambler (3–7)	16		(22.5)
Low-risk gambler (1–2)	16		(22.5)
Non-problem gambler (0)	31		(43.7)
**Age at onset of gambling (years old) (*N*** **= 97**^**b**^**)**			
< 20	33		(34.0)
≥ 20	64		(66.0)
**Form of gambling first engaged in (*N*** **= 97**^**b**^**)**			
Pachinko (EGM)	64		(66.0)
Pachislot (EGM)	5		(5.2)
Horse racing	15		(15.5)
Boat racing	5		(5.2)
Bicycle racing	1		(1.0)
Lottery	2		(2.1)
Mahjong, shogi, card betting	2		(2.1)
Other	3		(3.1)
**Whether they had close associations with those with gambling problems (*N*** **= 103)**			
Yes	36		(35.0)
No	67		(65.0)
**Close relatives and friends with gambling problems** **(multiple answers allowed) (*N*** **= 36)**			
Fathers	15		(41.7)
Mothers	6		(16.7)
Siblings	4		(11.1)
Spouse and/or cohabitants	1		(2.8)
Other relatives	3		(8.3)
Friends and/or significant others	19		(52.8)
**When they had gambled the most (*N*** **= 97**^**b**^**)**			
Before the first homelessness incident	67		(69.1)
Before, during, and after the first homelessness incident	15		(15.5)
After the first homelessness incident	15		(15.5)
**Reasons for not gambling as much as before (*N*** **= 86**^**c**^**)**			
I do not have the money for it	46		(53.5)
It costs a lot	13		(15.1)
Realized it was waste of money time/money	10		(11.6)
Lost interest	5		(5.8)
I would have become too into it	3		(3.5)
I just wanted to try it	2		(2.3)
I cannot win any more	2		(2.3)
I do not like the venue environment	1		(1.2)
Health reasons	1		(1.2)
No real reason	1		(1.2)
Other	2		(2.3)

GD: Gambling Disorder

SOGS: South Oaks Gambling Screening

PGSI: Problem Gambling Severity Index

EGM: Electronic Gaming Machine

^a^Those who had gambled in the past 12 month

^b^Six participants without gambling experience were not included

^c^Those who are not gambling as much as before

**Table 3:** Participants’ gambling activities in their lifetimes (when they gambled the most) and in the past year

	Participants who have ever gambled in their lifetimes (*n* = 97): When they gambled the most				Participants who have gambled in the past year (*n* = 71)	
**Gambling frequency**						
Everyday	37			(38.1)	1	(1.4)
1–6 times a week	46			(47.4)	35	(49.3)
1–3 times a month	9			(9.3)	24	(33.8)
< Once a month	5			(5.2)	11	(15.5)
**Forms of gambling with most money spent**						
Pachinko (EGM)	46			(47.4)	25	(35.2)
Pachislot (EGM)	16			(16.5)	14	(19.7)
Horse racing	18			(18.6)	17	(23.9)
Boat racing	6			(6.2)	9	(12.7)
Bicycle racing	2			(2.1)	1	(1.4)
Motorbike racing	1			(1.0)	0	(0.0)
Lottery	2			(2.1)	3	(4.2)
Mahjong, shogi, card betting	4			(4.1)	1	(1.4)
Other	2			(2.1)	1	(1.4)
**Annual income (¥)**						
< 1,000,000	17			(17.5)	55	(77.5)
1,000,000–1,999,999	20			(20.6)	13	(18.3)
2,000,000–2,999,999	15			(15.5)	1	(1.4)
3,000,000–3,999,999	17			(17.5)	1	(1.4)
4,000,000–5,999,999	19			(19.6)	1	(1.4)
≥ 6,000,000	9			(9.3)	0	(0.0)

EGM: Electronic Gaming Machine

¥: Japanese Yen

Among those with a potential GD in their lifetimes (*n* = 45), 68.9% (*n* = 31) were aware that they had lost or almost lost someone and/or something important to them at home, work, or school because of their gambling. However, only 15.6% (*n* = 7) of the respondents had sought help regarding their debt and gambling problems from a specialist or counselor.

### Associations between Homelessness and Gambling Disorder

Table 4 and Supplementary File 2 show a comparison of participants’ characteristics, with or without potential lifetime GD based on SOGS criteria, according to the bivariate logistic regression analysis. In this first step of our multivariable logistic regression analysis, eight variables were significant at *p* ≤ .10, including “previously married,” “≥20 years since the first incidence of homelessness,” “≥5 homelessness episodes,” “experience of using social support,” “early gambling onset,” “close relatives and friends with gambling problems,” “current smoking,” and “potential alcohol dependence.” The age of gambling initiation varied between those with GD (median = 18, M ± SD = 18.4 ± 6.41, range = 12–56) and those without (median = 18, M ± SD = 20.4 ± 6.26, range = 10–40), at *p* < .05. In the second step of our regression model, we entered variables selected beforehand, which included smoking, alcohol dependence, imprisonment history, and the variables with a significance level of *p* ≤ .10 in the first step. Younger age was also selected as one of the important variables but not included in the multivariable logistic regression model because only two of our participants were younger than 30 years. “Experience of using social support,” “current smoking,” “potential alcohol dependence,” and “have been imprisoned” were removed from the final model (*p* > .10). Table 5 shows the results of the multivariable logistic regression model, the *p*-values of which were all statistically significant. Marital status was only marginally associated with GD (AOR: 3.13, 95% CI: 1.00–9.81, *p*-value: 0.0497). Finally, a sensitivity analysis, in which we entered all the covariates was conducted. The resulting coefficients were similar and overlapping with those that we found with our primary approach in listed Table 5 (Supplementary File 3).

**Table 4:** Bivariate analysis of characteristics of homeless men with or without potential gambling disorder in their lifetimes (*N* = 103)

	With GD	Without GD	OR		95% CI		*p-*value
	*n* = 45	*n* = 58					
**Age (years)**							
< 50	6 (13.3)	12 (20.7)	0.59		0.20–1.72		0.333
≥ 50	39 (86.7)	46 (79.3)	Ref.				
**Educational status**							
Middle school graduate	23 (51.1)	29 (50.0)	1.05		0.48–2.28		0.911
High school graduate or higher	22 (48.9)	29 (50.0)	Ref.				
**Marital status**							
Never married (Never married, unmarried partner)	28 (62.2)	49 (84.5)	Ref.				
Previously married (divorced, widowed)	17 (37.8)	9 (15.5)	3.31		1.30–8.39		0.010
**Time elapsed since the first homelessness incidence (years)**							
< 10	14 (31.1)	29 (50.0)	Ref.				
10–19	12 (26.7)	18 (31.0)	1.38		0.52–3.64		0.514
≥ 20	19 (42.2)	11 (19.0)	3.58		1.34–9.52		0.011
**Number of homelessness episodes in lifetime**							
1	17 (37.8)	32 (55.2)	Ref.				
2–4	15 (33.3)	22 (37.9)	1.28		0.53–3.10		0.579
≥ 5	13 (28.9)	4 ( 6.9)	6.12		1.73–21.69		0.005
**Experience of using social support in lifetime**							
Yes	32 (71.1)	30 (51.7)	2.30		1.01–5.24		0.046
No	13 (28.9)	28 (48.3)	Ref.				
**Age at onset of gambling (years old)** ^**a**^							
< 20	35 (77.8)	29 (50.0)	2.78		1.14–6.76		0.023
≥ 20	10 (22.2)	23 (39.7)	Ref.				
**When they had gambled the most** ^**a**^							
Before the first homelessness incident	30 (66.7)	37 (63.8)	Ref.				
Before, during, and after the first homelessness incident	7 (15.6)	8 (13.8)	1.08		0.35–3.32		0.894
After the first homelessness incident	8 (17.8)	7 (12.1)	1.41		0.46–4.33		0.549
**Annual income when gambling the most (¥)** ^**a**^							
< 2,000,000	16 (35.6)	21 (36.2)	Ref.				
2,000,000–3,999,999	14 (31.1)	18 (31.0)	1.02		0.39–2.65		0.966
≥ 4,000,000	15 (33.3)	13 (22.4)	1.51		0.56–4.06		0.410
**Form of gambling when gambling the most** ^**a**^							
EGMs (Pachinko or Pachislot)	32 (71.1)	30 (51.7)	1.81		0.77–4.21		0.170
Other	13 (28.9)	22 (37.9)	Ref.				
**Whether they had close associations with those with gambling problems**							
Yes	26 (57.8)	10 (17.2)	6.57		2.66–16.19		< 0.0001
No	19 (42.2)	48 (82.8)	Ref.				
**Current smoking behavior**							
Yes	40 (88.9)	37 (63.8)	4.54		1.55–13.28		0.004
No	5 (11.1)	21 (36.2)	Ref.				
**Current alcohol use (AUDIT score)**							
Potential alcohol dependence (15–40)	12 (26.7)	5 ( 8.6)	6.00		1.68–21.48		0.006
Hazardous use (8–14)	7 (15.6)	8 (13.8)	2.19		0.63–7.65		0.220
No risk (1–7)	16 (35.6)	23 (39.7)	2.00		0.75–5.35		0.168
Non-drinker (0)	10 (22.2)	22 (37.9)	Ref.				
**Imprisonment history in lifetime**							
Yes	10 (22.2)	7 (12.1)	2.08		0.72–5.99		0.169
No	35 (77.8)	51 (87.9)	Ref.				

GD: Gambling Disorder (South Oaks Gambling Screening score ≥ 5)

EGMs: Electronic Gaming Machines

AUDIT: Alcohol Use Disorders Identification Test

OR: Odds Ratio

CI: Confidence Interval

^a^Six participants without gambling experience were not included

**Table 5:** Multivariate analysis of characteristics of homeless men with or without potential gambling disorder in their lifetimes (*N* = 97^a^)

	AOR	95% CI	*p*-value
Previously married (divorced, widowed)	3.13	1.00– 9.81	0.0497
≥ 20 years since the first homelessness incidence	4.97	1.50–16.45	0.009
≥ 5 homelessness episodes	4.51	1.06–19.26	0.042
Early gambling onset (< age 20)	4.69	1.46–15.07	0.010
Had close relatives/friends with gambling problems	5.85	1.96–17.41	0.002

Potential gambling disorder (South Oaks Gambling Screening score ≥ 5)

AOR: Adjusted Odds Ratio

CI: Confidence Interval

^a^Those without gambling experiences (*n* = 6) are not included in the analysis

## Discussion

This is the first epidemiological study in Japan investigating GD among homeless men. First, as in previous international studies, this study found a high prevalence of GD in Japanese homeless men. The lifetime prevalence in our study was 43.7% (*n* = 45) and 3.9% (*n* = 4) in the past year, much higher than the prevalence rates for men in 2017 JNSG, which were 6.7% and 1.5%, respectively (Higuchi & Matsushita, 2017). Compared with studies in other countries such as Canada and the United States, where lifetime prevalence was reported to be 10% (Matheson et al., 2014) and 12.0% (Nower et al., 2015), respectively, the lifetime prevalence among homeless people in our study was especially high. However, fewer Japanese were identified as “high-risk gamblers” in the past year using the PGSI (7.8%) than in the United Kingdom—at 11.6% (Sharman et al., 2015) and 23.6% (Sharman et al., 2016)—and Poland at 11.3% (Wieczorek et al., 2019). These differences in problem gambling prevalence rates among homeless populations may result from several factors, including the prevalence of GD in each country; cultural context of gambling; definition and characteristics of the homeless subgroups; and differences in sampling methods, response rates, scales used, and order of screens (Griffiths, 2015; Stevens & Young, 2008; Williams et al., 2012).

Second, this study found factors associated with lifetime GD such as exceeding “more than 20 years since the first incidence of homelessness” and having “experienced homelessness five or more times.” Findings in previous studies concerning a potential relationship between a long or frequent history of homelessness and GD were inconsistent (Nower et al., 2015; Shaffer et al., 2002; Wieczorek et al., 2019). Shaffer et al. (2002) reported that those who were pathological gamblers with substance use disorders tended to have become homeless at a younger age and experience homelessness more often than non-pathological gamblers. Notably, the causality between gambling addiction and homelessness in our study is unclear due to our cross-sectional design. However, GD may make it difficult for people to overcome homelessness or may facilitate chronic and episodic homelessness. Of the total sample of homeless, 68.9% (*n* = 71) had gambled in the past year, and one-third of them (*n* = 24) were found to be at moderate to high risk of problem gambling, according to the PGSI scores. Moreover, approximately half of the lifetime gambling participants cited that the reason they no longer gambled as much as before was the lack of money. Therefore, the low prevalence of GD in the past year shown in our study may be due to both spontaneous recovery from GD and economic limitations, owing to homelessness temporarily restricting gambling to levels that fail to meet diagnostic criteria for GD.

In this study, current alcohol dependence and smoking were not associated with lifetime GD. This is despite higher rates of smoking and alcohol dependence in those with GD than in those without it. Associations between alcohol and other substance use disorders and GD are well-known (Lorains et al., 2011), and high proportions of nicotine, alcohol, or any other substance dependence have been reported among homeless people with gambling problems (Nower et al., 2015; Sharman et al., 2016). A potential reason for GD not being associated with alcohol dependence and smoking in our results may be our exclusion of inebriated people from participation. This may have resulted in lower participation by those with alcohol problems, which may have affected the results. Furthermore, the 95% CIs were very wide, suggesting insufficient power, possibly leading to a Type 2 error. However, in a study targeting British homeless people, alcohol and substance use disorders were also not significantly associated with past-year problem gambling (Sharman et al., 2016). Exhibiting multiple addictions is a not infrequent issue. Some addictive behaviors do not necessarily coexist, and others may arise during withdrawal from a specific addictive behavior. Low income might limit access to and temporarily reduce the frequency of or expenditure on addictive behaviors.

When homeless people with GD aim to rebuild and stabilize their lives, the presence or resurgence of gambling problems and/or alcohol addiction are potential risks of recurrent homelessness. Although nearly 70% of participants in this study with GD were aware of the negative effects of gambling, 85.7% had never sought help. Homeless people tend to experience multiple and complex problems, and gambling problems may be overlooked or triaged due to reduced priority (Vandenberg et al., 2021). Therefore, as part of a comprehensive support system for homeless individuals, screening for gambling activities and their negative impact might be useful.

This study found that “early gambling onset” and having “close relatives and friends with gambling problems” were associated with GD. These factors were also reported in previous studies that targeted people who were not homeless (Black et al., 2013; Mann et al., 2017; Mazar et al., 2018; Shaw et al., 2007; Volberg, 1994). In Japan, Pachinko and Pachislot EGMs are popular forms of gambling, mainly used by men, and are legal for those older than 18 years. Among our participants, EGMs were reported as the most used in the past and the present. The proportion of those who had gambled in the past year was still high, at 68.9%. Preventive measures may need to include individuals with GD, those who are close to individuals with GD, and recreational gamblers.

### Limitations

This study has some limitations. First, the sample was limited in its representation, and the findings may not be generalizable to the entire homeless population in Japan. Our participants were only men using the shelters. Moreover, there is a high prevalence of psychological disorders and intellectual disabilities among homeless people (Morikawa et al., 2011; Nishio et al., 2015; Okamura et al., 2015; Okuda, 2010), and those with severe mental illness, including alcohol use disorder and intellectual disabilities, were not in scope in this study. However, because homeless subgroups are a diverse and hard-to-reach population, we consider this study’s setting suitable for an epidemiological study.

Second, potentially important variables were not measured. High rates of mental health disorders are reported in both those with GD and the homeless population. However, we used the same questionnaire as the JNSG 2017. In the present study setting, we determined that screening participants’ mental disorders using valid and reliable methods was not possible. Future research should explore the association between GD and other mental disorders among homeless population in more detail.

Third, 95% CIs of ORs and AORs were wide, indicating low power and uncertainty in the current study’s estimates. This may be partly due to our approach to examine relationships through ORs as some cells in the 2*2 tables had small sample sizes. Given the skewed distributions of some variables, in order to increase interpretability, we prioritized analyses with ORs. A sensitivity analysis using the original continuous scales confirmed the findings with ORs. We gained a unique opportunity in this study, but recruitment remained difficult among this hard-to-reach population. Effect sizes and 95% CIs of any correlates identified should be interpreted with caution. Furthermore, as this is a cross-sectional study, the results do not demonstrate causality between GD and homelessness or between GD and the associated factors.

Finally, gambling, alcohol problems, and imprisonment history were self-reported and may therefore be underreported. People with addictive disorders tend to underestimate their problems (Suurvali et al., 2009), and homeless people have greater difficulties disclosing their gambling problems (Holdsworth & Tiyce, 2012). Additionally, nine participants with a long history of homelessness were excluded because they had difficulty recalling their personal history related to homelessness. Homeless people have been shown to struggle with memory and other cognitive impairments (Ennis et al., 2015). This type of response bias is inherent to any study with homeless populations, and we determined that removing egregious instances on unreliable reporting was the best approach to adjust for such bias.

## Conclusions

This study provided initial epidemiological information on GD among homeless men in Japan. Overall, GD was found to be more prevalent among our sample than in the general population. For those with a lifetime of GD, more time had elapsed since the first incidence of homelessness, and episodes of homelessness were more frequent than among those without GD. Additionally, homeless men with GD were more likely to have started gambling at a younger age and have close relatives and friends with gambling problems than those without GD.

When homeless people with GD aim to rebuild and stabilize their lives, the presence or resurgence of gambling problems poses a potential risk of being homeless again. Homeless people often experience a combination of problems; thus, support for addressing gambling problems needs to be included in a comprehensive support program. Preventive measures should be taken at the population level and should include not only those with GD but also those with recreational gambling behaviors and their social networks.

## Electronic supplementary material

Below is the link to the electronic supplementary material.

Supplementary Material 1
