# Supplementary material for: Prevalence of gambling disorder and its correlates among homeless men in Osaka city, Japan
Source: J Gambl Stud. 2022 Jun 15;39(3):1059–76. doi: 10.1007/s10899-022-10121-x (PMC10397130; doi:10.1007/s10899-022-10121-x)
Supplement: Supplementary file 1 — Supplementary Material 1 [file 10899_2022_10121_MOESM1_ESM.docx]

**Supplementary File 1.** Distribution of continuous variables on participants’ characteristics and gambling experience (*N*=103)

|  | Median | Mode | Range | Shapiro-Wilk test | *ｐ*-value |
| --- | --- | --- | --- | --- | --- |
| Age (years) | 58 | 58 | 30 - 73 | 0.975 | 0.0510 |
| Time elapsed since the first homelessness incidence (years) | 10 | 10 | <1 - 40 | 0.936 | <.0001 |
| Number of homelessness episodes (times) | 2 | 1 | 1 - 30 | 0.515 | <.0001 |
| Income in December 2018 (¥) | 40,000 | 30,000 | 0-1,500,000 | 0.914 | <.0001 |
| AUDIT score (0-40) | 3 | 0 | 0 - 40 | 0.771 | <.0001 |
| PGSI score (0-27) ^a^ | 0 | 0 | 0 - 12 | 0.680 | <.0001 |
| Age at onset of gambling (years) ^a^ | 18 | 18 | 10 - 56 | 0.686 | <.0001 |

¥: Japanese Yen

AUDIT: Alcohol Use Disorders Identification Test

PGSI: Problem Gambling Severity Index

^a^ Six participants without gambling experience were not included.

**Figure.** Histograms for these continuous variables

Age (years)

Time elapsed since the first homelessness incidence (years)

Number of homelessness episodes (times)

Income in December 2018 (¥)

AUDIT score (0-40)

PGSI score (0-27)

Age at onset of gambling (years)

**Supplementary File 2.** Bivariate analysis of continuous variable (“age” and “time elapsed since the first homelessness incidence”) using Mann-Whitney U testof homeless men by with or without potential gambling disorder in their lifetimes (*N*=103)

|  | With GD (n=45) | | | |  | | Without GD (n=58) | | |  | |
| --- | --- | --- | --- | --- | --- | --- | --- | --- | --- | --- | --- |
|  | Median | Mean±SD | Range |  | | Median | | Mean±SD | Range | *p*-value |  |
| Age (years) | 58.0 | 57.5± 7.42 | 40 – 72 |  | | 58.5 | | 57.0±9.25 | 30 – 73 | 0.995 |  |
| Time elapsed since the first homelessness incidence (years) | 15.0 | 15.7±10.39 | <1 – 36 |  | | 9.5 | | 11.3±9.12 | <1 – 40 | 0.026 |  |

GD: Gambling Disorder (South Oaks Gambling Screening score ≥ 5)

As shown in Supplementary File 1, the distribution of “number of homelessness episodes,” “AUDIT score,” and “PGSI score” were extremely skewed towards zero and the smallest categories (1 time, score 0, and score 0, respectively) were the most frequent. Therefore, we calculated ORs with these as references.

“The age at onset of gambling” was not normally distributed either (Shapiro-Wilk test=0.686, p<.0001). We categorized the age of gambling onset as “< 20 years” and “≥ 20 years.” The cut-off age is the legal age of gambling in Japan.

As results of the Mann-Whitney U test for the remaining variables (“age” and “time elapsed since the first homelessness incidence”), only “time elapsed since the first homelessness incidence” was statistically significantly different between those with or without potential GD in their lifetime (Supplementary File 2).

**Supplementary File 3.** Multivariate analysis of characteristics of homeless men with or without potential gambling disorder in their lifetime using all the variables (n = 97^a^)

|  | AOR | 95% CI | *p*-value |
| --- | --- | --- | --- |
| < Age 50 | 0.86 | 0.16 – 4.65 | 0.861 |
| Middle school graduate | 0.69 | 0.21 – 2.28 | 0.542 |
| Previously married (divorced, widowed) | 4.12 | 1.05 – 16.23 | 0.043 |
| ≥ 20 years since the first homelessness incidence | 5.73 | 1.38 – 23.80 | 0.016 |
| ≥ 5 homelessness episodes | 3.17 | 0.54 – 18.49 | 0.200 |
| Had used public social homeless support services | 2.14 | 0.58 – 7.93 | 0.254 |
| Early gambling onset (< age 20) | 7.36 | 1.71 – 31.68 | 0.007 |
| Had close relatives/friends with gambling problems | 6.90 | 1.92 – 24.84 | 0.003 |
| Had gambled the most before the first homelessness incident | 1.58 | 0.40 – 6.25 | 0.511 |
| < 2,000,000 of annual income when gambling the most | 2.17 | 0.57 – 8.24 | 0.256 |
| Played EGMs when gambling the most | 1.16 | 0.30 – 4.51 | 0.833 |
| Currently smoking | 1.94 | 0.44 – 8.65 | 0.384 |
| Potential alcohol dependence (AUDIT score 15–40) | 2.54 | 0.50 – 12.89 | 0.261 |
| Having imprisonment history | 0.50 | 0.09 – 7.29 | 0.431 |

EGMs: Electronic gaming machines

AUDIT: Alcohol Use Disorders Identification Test
